# Low transmission risk of African swine fever virus between wild boar infected by an attenuated isolate and susceptible domestic pigs

**DOI:** 10.3389/fvets.2023.1177246

**Published:** 2023-08-10

**Authors:** Aleksandra Kosowska, Jose A. Barasona, Sandra Barroso-Arévalo, Luisa Blondeau Leon, Estefanía Cadenas-Fernández, Jose M. Sánchez-Vizcaíno

**Affiliations:** ^1^VISAVET Health Surveillance Center, Complutense University of Madrid, Madrid, Spain; ^2^Department of Animal Health, Faculty of Veterinary Medicine, Complutense University of Madrid, Madrid, Spain

**Keywords:** African swine fever, transmission, wild boar, domestic pig, interspecific, interactions, attenuated isolate, virulent isolate

## Abstract

African swine fever (ASF) is a lethal infectious disease that affects domestic and wild pigs. This complex virus has already affected five continents and more than 70 countries and is considered to be the main threat to the global swine industry. The disease can potentially be transmitted directly through contact with infectious animals, or indirectly by means of contaminated feed or environments. Nevertheless, the knowledge regarding the transmission patterns of different ASF virus isolates at the wildlife-livestock interface is still limited. We have, therefore, assessed the potential transmission of an attenuated ASF virus isolate between infectious wild boar and directly exposed domestic pig. We registered 3,369 interspecific interactions between animals, which were brief and mostly initiated by wild boar. The major patterns observed during the study were head-to-head contact owing to sniffing, thus suggesting a high probability of pathogen transmission. However, only one of the five domestic pigs had a short period of viremia and became serologically positive for ASF virus antibodies. It was additionally discovered that the wild boar did not transmit the virulent virus isolate to the domestic pigs, which suggests that the presence of attenuated ASF virus isolates in affected areas may control the spreading of other more virulent isolates. These outcomes may help make decisions related to large-scale targeted management actions against ASF in field conditions.

## 1. Introduction

African swine fever (ASF) is a devasting hemorrhagic viral disease that affects the *Suidae* family and is harmful to domestic and wild pigs of all ages and sexes (1). The disease is caused by the African swine fever virus (ASFV), which is a large, enveloped DNA virus, and belongs to the family *Asfaviridae*. ASFV infection may appear in susceptible populations in a wide variety of clinical forms, from subclinical to severe hemorrhagic disease with a high lethality often from 90 to 100% (2). In Europe, the control of the disease is based on its rapid diagnosis and the implementation of strict sanitary measures since commercial vaccines and effective therapies are not available. The appearance of new ASF outbreaks in ASF-free countries leads to export restrictions on live animals and their products, thus triggering, huge economic losses.

ASFV, which belongs to the genotype II, reappeared in Georgia in 2007. It subsequently affected Transcaucasian countries and quickly spread to the Russian Federation, reaching European Union countries in 2014. In Europe, ASF is currently present in Lithuania, Poland, Latvia, Estonia, Moldova, Bulgaria, Hungary, Romania, Slovakia, Serbia, Greece, Germany, North Macedonia, Italy, and recently, Ukraine, Bosnia and Herzegovina and Croatia. Effective outbreak management in the Czech Republic and Belgium followed, resulting in these countries being declared ASF-free in 2019 and 2020, respectively (3). Nevertheless, after an absence of more than 3 years, ASF has re-emerged in the Czech Republic, where the carcasses of infected wild boar have been found (4). In August 2018, the ASF crisis expanded throughout Asia, where the first outbreak of ASF was reported in the world's largest pig producing country, China (5). The virus has since spread rapidly and has, to date, affected neighboring countries such as Mongolia, Vietnam, Cambodia, Hong Kong, the Democratic People's Republic of Korea, Laos, Myanmar, Philippines, the Republic of Korea, Timor-Leste, Indonesia, India, Malaysia, Thailand and Papua New Guinea in Oceania (4). The most recent update from the World Organization for Animal Health (WOAH) confirmed new cases of ASF in the Western hemisphere for the first time in ~40 years. The ASF-positive pig samples were confirmed in the Dominican Republic and Haiti as a part of a cooperative surveillance program between the United States and the Dominican Republic (4).

The epidemiology of ASF is complex and varies according to the environment, types of production system, the presence or absence of wild pigs, competent tick vectors, and human behavior (1). The circulation of the virus in the natural ecosystem has, in the last decade, developed into a self-sustained epidemiological cycle with the implication of the wild suids (6). The involvement of wild boar (*Sus scrofa*) population in ASFV maintenance, spread, and transmission is of particular concern on the European and Asian continents owing to its extensive presence in these territories (5, 7). All efforts directed toward ASF control should, therefore, consider the important role played by wild boar in pathogen transmission.

The clinical course of the ASFV infection depends on multiple variables, such as, the virulence of the virus and the individual immunological characteristics of the host (8–12). The principal routes of ASFV transmission are related to blood, excretions, secretions, or the carcasses of infected wild boar (13). It has been demonstrated that direct contact is an effective ASFV transmission route between infected and susceptible suids (9, 14, 15). With regard to the virulence of the virus, wild boar infected with virulent ASFV isolates developed serious clinical signs similar to those observed in domestic pigs, and died within seven to nine days (9, 16, 17). Furthermore, other less virulent ASFV isolates, such as Lv17/WB/Rie1 (genotype II) or NH/P68 (genotype I), have been shown to produce mild to absent clinical signs with transitory fever, and obtained good results as regards protective immunity against virulent isolates (18–21). These attenuated isolates are currently being evaluated in the UE-funded research project H2020 VACDIVA (Grant Agreement n° 862874) which focuses on the development and assessment of vaccine candidates as a safe and effective tool for wild boar and domestic pig populations. However, the use of live attenuated vaccines (LAVs) based on naturally attenuated virus isolates may, overall, entail a series of concerns related to the shedding of the vaccine virus, reversion to virulence, or the generation of new variants with wild-type viruses (22). These safety concerns of LAVs should be reduced to a minimum, and it is for this reason, that deletion mutants based on naturally attenuated virus isolates are currently the most promising options with which to control the spread of ASFV and reduce the risk of the devasting consequences of this disease for swine producers worldwide (23–25).

The issue with LAVs is their ability to occasionally transmit the attenuated virus to susceptible animals. The potential shedding of attenuated virus isolates such as Lv17/WB/Rie1 has already been investigated in domestic pigs (26) and wild boar (27). The results described in these studies suggest that the risk of oral shedding, which is the natural route of infection, is much lower with attenuated viruses than with highly virulent or moderate virulence isolates (15, 26). However, animals infected with attenuated isolates have demonstrated the capacity to transmit the virus to sentinel animals (19, 21). For instance, the attenuated virus Lv17/WB/Rie1 was transmitted to sentinel wild boar within 2 weeks (21), while NH/P68 was transmitted to sentinel pigs within 3 or 4 weeks after the initial exposure (19). In both cases, animals previously infected with these LAVs did not transmit the virulent challenge virus to sentinel animals. This demonstrated that susceptible animals were successfully protected by direct contact, which could have beneficial effects for control strategies (13, 28).

In the present study, we investigated the transmission rate of the attenuated ASFV isolate Lv17/WB/Rie1 between wild boar and domestic pigs. A group of sentinel domestic pigs was, therefore, exposed through direct contact with wild boar infected with the attenuated ASFV isolate. All details of the interspecific interactions between the two subspecies and their clinical consequences are described herein.

## 2. Material and methods

### 2.1. Animals

The experiment was carried out using four 4–5-month-old female and one male wild boar weighing 20–25 kg, and five castrated 2-month-old Large White breed male pigs, weighing 15–20 kg. The wild boar were obtained from a commercial farm in Sevilla, Spain, while the domestic pigs were from an authorized breeding farm in Segovia, Spain. The experiment was performed in biosafety level 3 (BSL-3) facilities at the VISAVET Health Surveillance Centre at the University Complutense of Madrid, Spain. Animal care, management and sampling procedures were conducted according to national and European regulations and the experimental protocol was previously approved by the Ethics Committee of the Complutense University of Madrid and the Community of Madrid (reference PROEX 159/19). The protocol included a detailed description of efforts to prevent and avoid the animals' unnecessary suffering, including humane endpoints and guidelines regarding euthanasia, following the EC Directive 2010/63/UE. All procedures were designed and performed by specially trained personnel and veterinarians (animal experimentation categories B, C, and D) following the Directive 2003/65/EC and Spanish laws RD53/2013. Guidelines for ARRIVE 2.0 for the care and use of laboratory animals were also followed.

Upon arrival, the animals were individually ear-tagged and acclimated for one week before the experiment began. Access to food and water was provided *ad libitum* throughout the study. These animals were not vaccinated against any pathogen and tested negative for ASFV and the main porcine pathogens in the region: *Mycoplasma hyopneunoniae, Mycobacterium bovis*, porcine reproductive and respiratory syndrome (PRRS) virus, porcine circovirus type 2.

### 2.2. ASFV isolates

The wild boar were infected using the attenuated non-hemoadsorbing p72 genotype II ASFV Lv17/WB/Rie1 isolate. This isolate has previously been tested on domestic pigs and wild boar, showing promising results in terms of effectiveness against the challenge with a highly virulent ASFV isolate, Armenia 2007 (20, 21, 29). The virus was grown for 7 days in porcine blood monocytes (PBM) and was collected as described previously by Barasona et al. (21). Viral titer was defined as the amount of virus causing cytopathic effects in 50% of infected cultures (TCID_50_/mL), estimated by employing immunoperoxidase staining (19).

The challenge virus employed was the virulent and hemadsorbing p72 genotype II ASFV Armenia 2007 (Arm07) isolate. The virus was propagated in PBM as previously described by Gallardo et al. (19), and the viral titer was defined as the amount of virus causing hemadsorption in 50% of infected cultures (HAD_50_/mL).

Both isolates were provided by the European Union Reference Laboratory (EURL) for ASF (CISA-INIA, Valdeolmos, Spain).

### 2.3. Wild boar infection

After the acclimatization period, the five wild boar were orally infected with a 10^4^ TCID_50_ dose of the attenuated ASFV Lv17/WB/Rie1 isolate. This infection dose demonstrated to be effective in previous studies where wild boar were orally inoculated (21, 29). Eighteen days after prime inoculation, these wild boar received a second dose of the ASFV Lv17/WB/Rie1 isolate with the same dose and route of administration. The five susceptible domestic pigs were housed jointly and exposed to direct contact with the infected wild boar in order to evaluate the transmission of the attenuated isolate. It was not possible to allocate more than two wild boar with all the susceptible pigs at the same time owing to space limitations in the pen. The order of the contact of the wild boar was determined randomly (Table 1).

**Table 1:** The allocation period of each wild boar with susceptible domestic pigs during the study (days post-infection).

**Wild boar ID**	**Days post-infection (dpi)**
WB 1	12–40
WB 2	40–74
WB 3	0–40
WB 4	40–74
WB 5	0–12

After the infection period of 42 days, all the wild boar were intramuscularly inoculated with 10 HAD_50_ of the virulent ASFV isolate, Arm07. The infection period, which could be understood as the period between the prime inoculation and the challenge was expressed in days post-infection (dpi) for the wild boar and days post-exposure (dpe) for the domestic pigs. The infected wild boar and susceptible domestic pigs were maintained together for 32 days post-challenge (dpc), a total of 74 days.

### 2.4. Interspecific interactions between wild boar and domestic pigs

**Figure 1:**
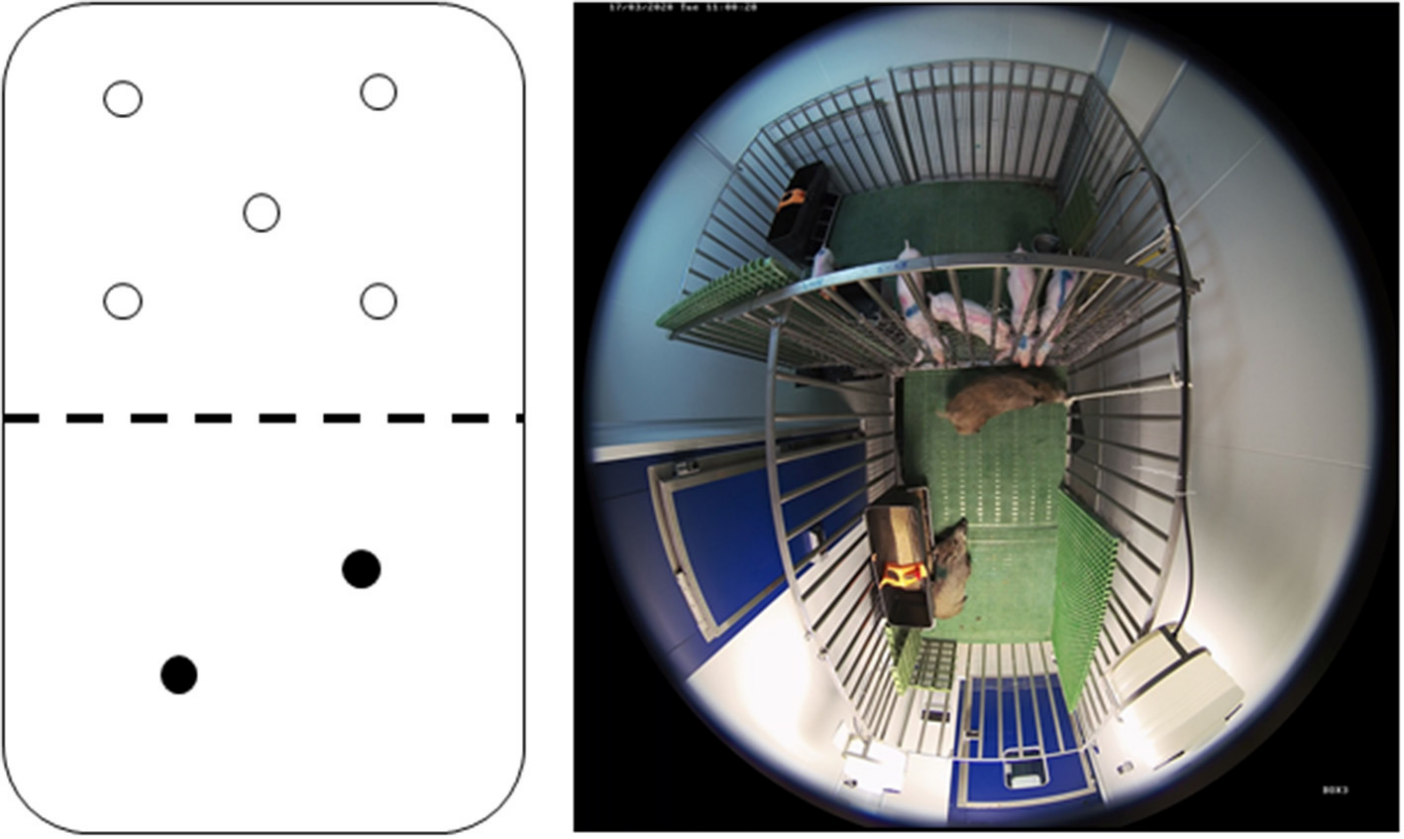
Schematic illustration and photo of the pen taken by a camera from the video surveillance system. Susceptible domestic pigs (transparent circle; *n* = 5) coming into contact with the infected wild boar (black circle; *n* = 2) through a metal livestock fence that separates the compartments of the pen.

The interactions between the infected wild boar and the susceptible pigs sharing the same pen were monitored by means of a video surveillance system (Hikvision iVMS-4200, Hikvision^®^, Hangzhou, China) throughout the study. Direct contact occurred through a metal livestock fence that separated the two compartments of the pen in order to prevent fights between these subspecies (Figure 1). During the experimental period, all details of interspecific interactions was specified using video surveillance. We registered the animals that participated in each interaction (subspecies and identification number), the type of interaction, and its duration. The type of interaction was considered at five levels: 1-simple approaches (<30 cm), 2-sniffing, 3-skin contact, 4-mucocutaneous contact, and 5-grooming or bites. The degrees of interaction levels 3, 4, and 5 (skin and mucocutaneous contact, grooming or bites) were considered high-risk contacts. The time of interaction was expressed in four time ranges: <30 s, 30 s - 1 min, 1 min - 5 min, more than 5 min. We also registered the number of animals involved in contact and which animal initiated the contact.

### 2.5. Clinical monitoring

The animals were observed daily throughout the trial in order to monitor their health status, by means of a video surveillance system and direct inspections carried out by veterinarians. Clinical signs, including rectal temperature, were expressed individually in terms of a quantitative clinical score (CS) specific to ASFV infection in domestic pigs (19, 30) and in wild boar (31). Fever was defined as a rectal temperature ≥40°C.

This CS considers nine parameters, which are rectal temperature, behavior, body condition, skin alterations, ocular/nasal discharge, swelling of joints, respiratory symptoms, digestive symptoms, and neurological symptoms. All clinical observations were recorded on a daily basis, with the exception of temperature, which was taken during the sampling (once a week during the infection period and twice a week during the challenge period), in order to minimize animal handling and stress.

Clinical evaluations were also monitored so as to ensure the animals' welfare. The humane endpoint was pre-defined as animals with a CS > 18, and animals with severe clinical signs (level 4) of fever, behavior, body condition, respiratory and digestive signs for more than two consecutive days were also included, following the standards described by Cadenas-Fernández et al. (31). Any animals undergoing unacceptable suffering without reaching the pre-defined humane endpoint were also euthanized on the basis of veterinarian criteria.

### 2.6. Sample collection, ASFV DNA, and antibody detection

Paired EDTA blood and sera were obtained from all the animals once a week during the infection period and twice a week during the challenge period.

Additionally, oral fluid and feces were collected from the wild boar in order to detect ASFV DNA. This was done by employing quantitative PCR (qPCR). Feces were collected using cotton swabs (Deltalab, Barcelona, Spain) and oral fluid was obtained using sponge swabs (ZIZNBA, Guangdong, China).

Viral DNA was extracted from each sample using the High Pure Template Preparation Mix Kit (Roche Diagnostics GmbH, Mannheim, Germany) according to the manufacturer's instructions. The detection of ASFV DNA from different types of samples (blood, oral fluid, feces) was performed using the Universal Probe Library (UPL) real-time quantitative PCR (qPCR) previously described by Fernández-Pinero et al. (32). A positive result for the qPCR was determined by identifying the threshold cycle value (cycle of quantification: Cq) at which a reporter dye emission appeared above the background within 40 cycles. Negative control samples were collected on day 0, which was the day of prime inoculation.

The sera samples were tested in order to detect antibodies. This was done using a commercial ELISA test (Ingenasa-Ingezim PPA Compac K3; Ingenasa, Madrid, Spain) and an indirect immunoperoxidase test (IPT).

At the end of the observation period (74 dpi/dpe), any surviving animals were anesthetized by means of an intramuscular injection of a combination of tiletamine-zolazepam (Zoletil^®^ 100 mg/ml, Virbac, France) and medetomidine (Medetor^®^, Virbac, France) (33), and were then euthanized by employing intravenous injection of T61^®^ (Intervet, Spain).

### 2.7. Statistical analysis

The records obtained from the video-surveillance monitoring of the trial, the sanitary results of the clinical inspections and laboratory analyses were unified in a dataset for a preliminary exploration. Overall, descriptive statistics were used for the evaluation of the interspecific interaction parameters and comparison to pathogen transmission findings. The relationships between the different categorical variables were assessed by using the Chi-square test (χ^2^) with a significance level of 95%. Continuous variables quantifying the number of contacts per species and individuals were assessed using the Kolmogorov-Smirnov test (KSt) to confirm the absence or presence of statistical normality. The Student's *t*-test and the Mann–Whitney *U*-test were used to detect differences between continuous variables according to KSt screening. The statistical evaluation of continuous variables concerning the occurrence of contacts and the degree of interaction was carried out using the Spearman rank correlation test. The statistical analysis was caried out using SPSS Statistics Version 25 (IBM Corporation, USA).

## 3. Results

### 3.1. Interspecific interactions between wild boar and domestic pigs

**Figure 2:**
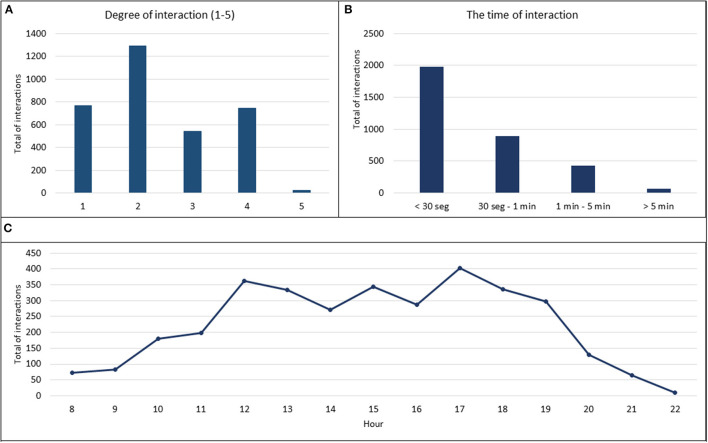
Results of interspecific interactions between infected wild boar and susceptible pigs. **(A)** The type of interaction was evaluated on a five degree scale: 1-simple approaches (< 30 cm), 2-sniffing, 3-skin contact, 4-mucocutaneous contact, and 5-grooming or bites, and the results were expressed in a total number of interactions. **(B)** The time of interaction was expressed in four time ranges and the results were expressed as the total number of interactions. **(C)** Daily activity profile of susceptible pigs and infected wild boar, expressed as the total number of interactions by an hour of the day.

Overall, we registered 3,369 interspecific interactions between the infected wild boar and the susceptible pigs. The major pattern observed during the study period was head-to-head contact and sniffing (38% of total interactions). We registered 39% of high-risk transmission contacts (degrees 3, 4, and 5) (Figure 2A). Interestingly, 60% of these high-risk contacts were initiated by the wild boar. With regard to the duration of interaction, the percentage of the appearance of contact was inversely proportional to the duration of contact (Spearman's correlation test R = −95%; *p* <0.001). In this respect, short periods of contact of <30 s predominated in most of the observations (59%). Only 2% of the interactions lasted 5 min or more, and were registered frequently when the animals fell asleep on either side of the metal fence (Figure 2B).

The daily activity register showed increased activity between 11:30 and 18:30 h with two peaks of interactions at 12 and 17 h (Figure 2C). This pattern was maintained as regards the interactions initiated by the wild boar and those initiated by the domestic pigs. The days with the highest daily activity were registered after the introduction of new wild boar into the pen, with an average number of registered interactions of 158 ± 45 in comparison to 64 ± 24 registered on a usual day.

With regard to which species favored the initiation of the interaction, 52% of the observations were initiated by the wild boar and 31% by the pigs. For the remaining percentage, it was not possible to identify which species initiated the interaction. In this respect, it was possible to observe a statistical difference with a major tendency for the wild boar to initiate interactions when compared to the domestic pigs (Student's *t*-test; *t* = 8.88; *p* < 0,001). Furthermore, almost half of the observations (49%) showed that the domestic pigs interacted in-group, while this was the case of the wild boar in only 16% of the total observations (Chi-square test; β = 860.6; *p* = 0.001). There are significant differences in the contact rate observed at the individual level. Wild boar WB 1 participated significantly more in interspecific contacts when compared with the other individuals (β = 19.9; *p* < 0.001). Pig P1 had significantly more interactions (both individual and in-group) then pigs P2 (β = 5.41; *p* = 0.02) and P4 (β = 6.32; *p* = 0.01). In addition, marginally significant differences were observed in the comparison between pig P1 and the other individuals (β = 3.68; *p* = 0.05).

### 3.2. Clinical and laboratory analysis

**Figure 3:**
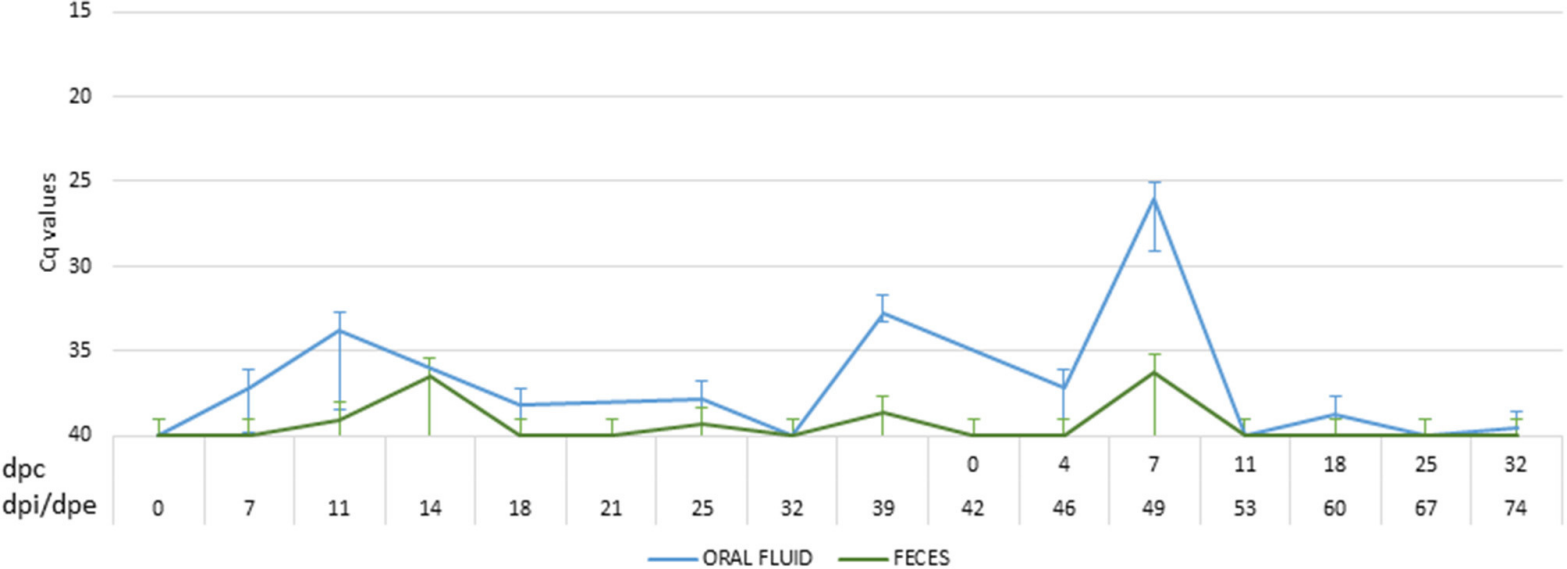
Mean of the DNA African swine fever virus (ASFV) load excreted by oral fluid (blue) and feces (green) of wild boar infected with Lv17/WB/Rie1 ASFV isolate. The viral load was expressed in cycles of quantification values (Cq) obtained by employing real-time PCR. The period between the prime inoculation and the challenge was expressed in days post-infection (dpi) for wild boar and days post-exposure (dpe) for domestic pigs, while the period after the challenge was expressed in days post-challenge (dpc).

**Figure 4:**
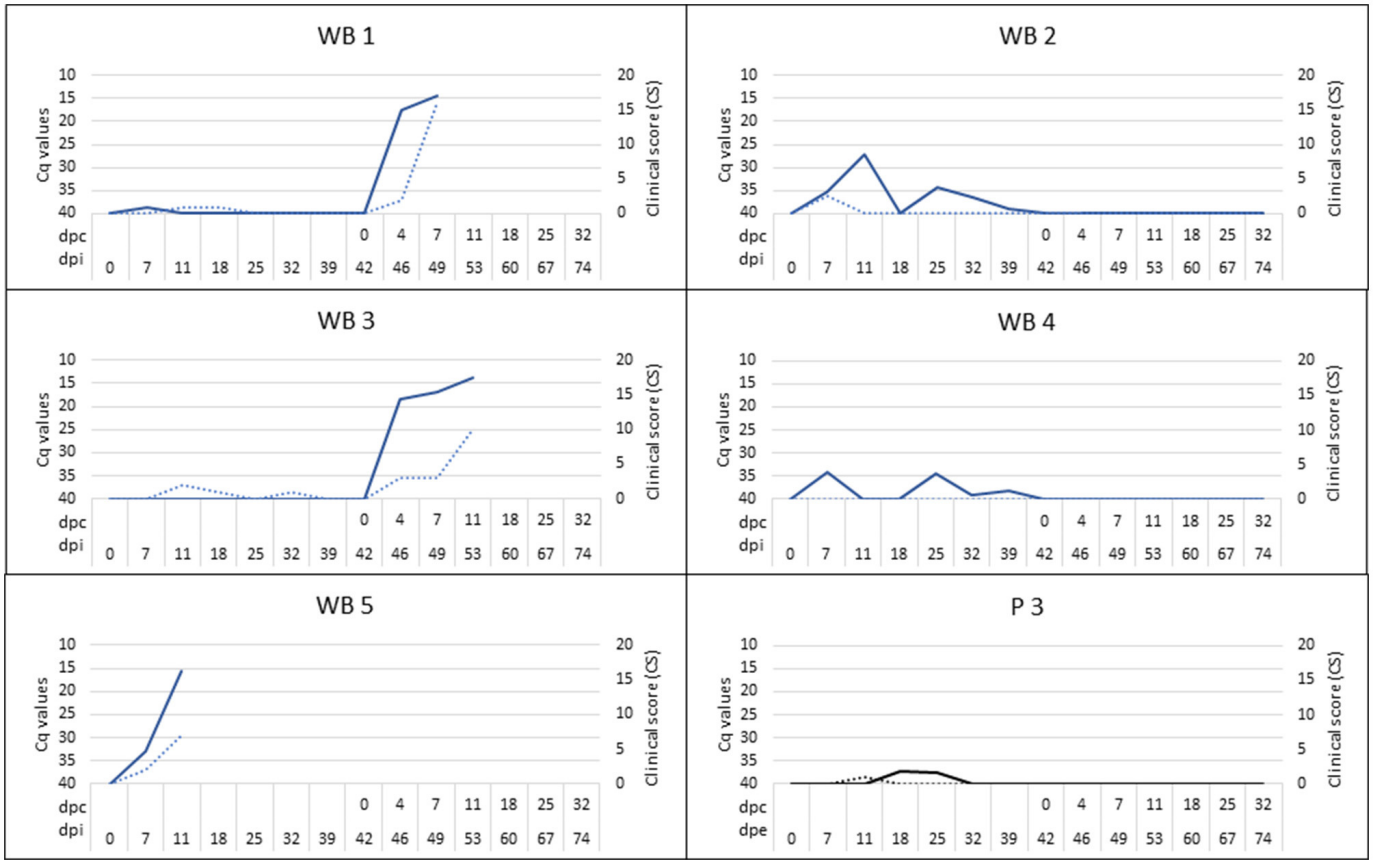
Clinical score (CS; dashed line) and viremia (continuous line) were determined by means of real-time PCR and expressed in cycles of quantification (Cq) for infected wild boar (WB 1-WB 5) and susceptible pig (P 3) exposed by direct contact. Four of the five susceptible pigs (P1, P2, P4, P5) did not have clinical signs or viremia. The period between the prime inoculation and the challenge was expressed in days post-infection (dpi) for wild boar and days post-exposure (dpe) for domestic pigs, while the period after the challenge was expressed in days post-challenge (dpc).

After coming into contact with the attenuated isolate, three of the five wild boar (60%) were successfully infected and had a positive antibody response, confirmed by ELISA and IPT, starting at 12 dpi. During the infection period, the wild boar had two clear peaks of viremia at 12 dpi (Cq = 33.66 ± 7.97) and 25 dpi (Cq = 37.13 ± 2.86), following prime and boost infection (Figure 4). In this period, three of the five wild boar had slight fever (40.00 ± 0.28°C) and occasionally lethargy, accompanied by viremia (Cq = 37.75 ± 4.23). Viral DNA was detected intermittently in wild boar excretions with a low viral load in oral fluid (Cq = 36.98 ± 3.89) and feces (Cq = 39.34 ± 1.22) (Figure 3).

**Figure 5:**
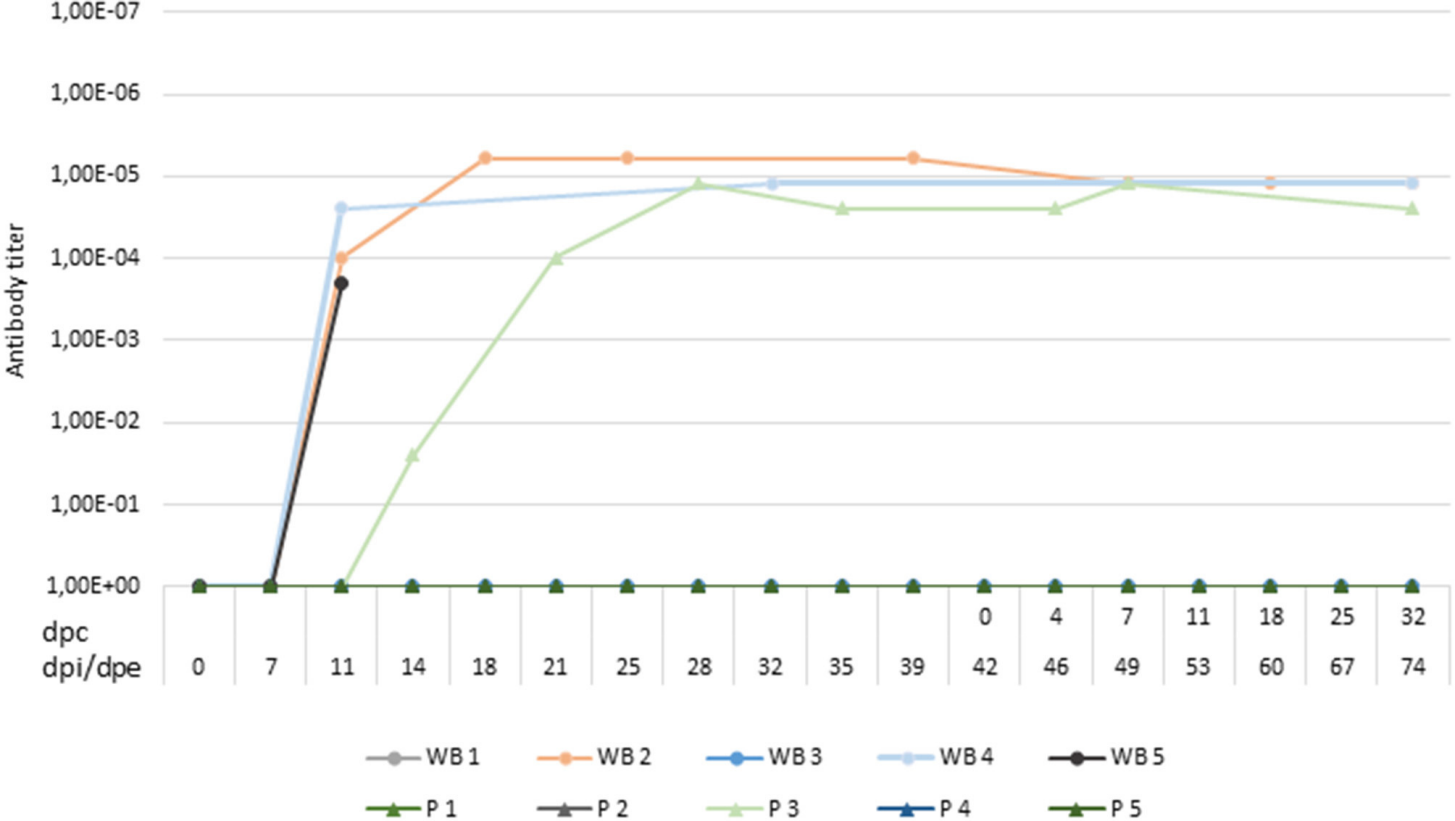
Titers of antibodies against African swine fever virus in wild boar infected with Lv17/WB/Rie1isolate (WB1, WB2, WB3, WB4, WB5) and domestic pigs exposed through contact with the infected animals (P1, P2, P3, P4, P5). A positive antibody response was not observed in two of the five wild boar infected. Titers were determined using the indirect immunoperoxidase test. The period between the prime inoculation and the challenge was expressed in days post-infection (dpi) for wild boar and days post-exposure (dpe) for domestic pigs, while the period after the challenge was expressed in days post-challenge (dpc).

One of the infected wild boar (WB 5), which appeared to be clinically healthy, but proved to be viremic (Cq = 27.11), did not recover after a handling procedure and anesthesia and was euthanized at 12 dpi. At the moment of death, this wild boar had significant viremia (Cq = 15.45; Figure 4), fever (40.9°C), and a low titer of ASFV-specific antibodies, which was detected using IPT (Figure 5). This animal was replaced with another in order to always maintain two wild boar in contact with the susceptible pigs.

At 14 dpe, two of the five susceptible pigs underwent a slight increase in body temperature (40.2°C) and suffered from slight depression. Viral DNA was detected in the blood (Cq = 37.38) of one of them (P3), which was maintained only in the next sampling. This pig also had a positive antibody response based on ELISA and IPT, reaching a high titer of antibodies, similar to those obtained for the infected wild boar (Figure 5).

Two of the infected and successfully protected wild boar, which were allocated with susceptible domestic pigs, survived the challenge carried out with the virulent ASFV isolate, Arm07. We did not detect fever or any other clinical signs that could be compatible with ASFV infection, and no ASFV DNA was detected in their blood. All of the susceptible pigs survived the challenge period and did not have any clinical signs of infection or fever, and no ASFV DNA was detected in their blood. One of the five susceptible pigs (P3) remained positive for the detection of specific antibodies against ASFV after the challenge period.

## 4. Discussion

Understanding the transmission mechanisms and sources of infection of ASFV has become a research priority in the current Eurasian context, in which wild boar play a key role in the epidemiology of the infection (7). In addition to this, with regard to the urgent need to develop and evaluate of multiple attenuated vaccine candidates in an attempt to prevent the advance of this infection (24), exploring the transmission capacity of these isolates is an important advance as regards deciphering gaps in research. This is, to the best of our knowledge, the first study to assess the potential transmission of an attenuated ASFV between wild boar and domestic pigs under experimental conditions. We have specifically, evaluated the spread of the ASFV Lv17/WB/Rie1 isolate in susceptible domestic pigs exposed by contact through a simple partition, simulating a livestock fence, with infected wild boar. Overall, we have determined high contact rates among the groups studied, suggesting a high probability of pathogen transmission. However, two of the susceptible pigs showed signs of infection, and only one had a short period of viremia and became serologically positive for ASFV antibodies. The results confirmed that the transmission capacity of this attenuated ASFV isolate is relatively lower than that of other genotype II isolates previously studied (14).

In this work, we have focused on carrying out a detailed assessment of direct interactions between wild boar and domestic pigs in order to understand potential cross-species pathogen transmission. Extensive monitoring, with more than 890 h of video-surveillance, has been performed to obtain detailed interactions between animals. This monitoring effort made it possible not only to discover interactions at a quantitative level but also to provide additional qualitative information related to the intensity, type and duration of these interactions. In general, most of the contacts were brief, with periods of <30 s, during which there was no aggressive behavior but rather curiosity. This result supports other studies carried out in the field, in which mostly indirect interactions between these subspecies were observed, and when direct interactions existed, they were for short periods (34, 35). Despite the brevity of these interactions, the relative risk of transmission could be high owing to a high proportion of oral and nasal mucosal contacts. In the case of highly or moderately virulent isolates of ASFV, this would be sufficient to achieve effective transmission (9, 16). However, in the case of the attenuated isolate studied, this has been observed to a lesser extent. We have also registered a low number of longer interactions (> 5 min), but these contacts corresponded to the occasions on which the animals were resting on either side of the metal fence, which was likely owing to space constraints inherent to an experiment in controlled laboratory conditions. Interestingly, the wild boar initiated contact through the fence more frequently than the pigs, despite the lower proportion of wild boar kept simultaneously in the pen throughout the experiment for animal welfare reasons. In this respect, the wild boar were more predisposed to a single contact as an individual, while the pigs tended to make contact in a group. This could be explained by the natural social behavior of pigs, based on the imitation of certain patterns observed in other individuals (36). The daily pattern of interaction by hours determined a higher risk between 11:30h and 18:30h, which is consistent with field studies in temperate environments in seasons with low thermal fluctuation, such as spring and autumn in Mediterranean scenarios (37, 38).

These results should be considered with caution, because they may be highly influenced by the experimental conditions of our study, which could differ from real scenarios. In the natural environment, interspecific interactions can be monitored through the use of tracking technologies such as GPS-collars, proximity loggers, or photo-trapping. As mentioned previously, these field studies indicate that direct interactions between livestock and wildlife occur rarely, and animals most often interact indirectly during the common use of water sources and supplementary feeding points (37–40). Although the data regarding contacts obtained in this study may have been altered by the experimental design, and there may be many factors affecting these interaction patterns, our results suggest that this may be a first approach with which to predict pathogen transmission between wild boar and domestic swine, and its applicability to reduce the consequences of these interspecific interactions. These results should be verified and extended in further field studies under controlled conditions and with a larger number of groups and animals.

With regard to the individual differences observed, the male wild boar (WB 1) had a higher number of interactions when compared to the females, which is mainly explained by a higher activity described in behavioral studies for this gender, along with sexual interest as a sign of early puberty. Furthermore, one of the susceptible domestic pigs (P1) showed a significantly higher number of interactions than the other pigs. However, this increase in interactions was not sufficient to cause infection with the attenuated ASFV isolate, despite increased exposure to infected wild boar. The results obtained in this study suggest a very sporadic transmission of the ASFV Lv17/WB/Rie1 isolate, which means that a higher transmission risk pathway would be needed to initiate transmission with this isolate from the wild boar to the domestic pig than would be observed with simple oro-nasal contact. This observation confirms that this attenuated isolate has the potential to be disseminated. However, the shedding pattern is limited, as also confirmed by previous studies (21, 26, 27). In this respect, the transfer/consumption of blood from an infected animal to a susceptible animal may be the cause of transmission, as previously suggested in the case of isolates of moderate to low virulence (26, 41). We can not rule out that blood consumption (e.g., through a bite from a viremic wild boar) occurred only in the case of the one pig (P3) that became infected with this attenuated isolate. This single infected pig, together with the other boars, was not able to spread the disease to the rest of the sentinel pigs beyond day 14 dpe, thus suggesting a null capacity to maintain carriers with transmission capacity. This outcome coincides with those of two long-term studies, which determined that animals infected with moderately virulent ASFV did not transmit the virus to commingled sentinel pigs after clinical recovery from ASF (42, 43).

Another important result of this study is the fact that the wild boar were successfully protected with the administration of the Lv17/WB/Rie1 isolate, did not transmit the virulent virus isolate to the susceptible pigs for 32 days, and survived the challenge with the virulent ASFV isolate, Arm07, without ASF-compatible pathognomonic signs or associated viremia. This observation could indicate that the infection of wild boar or the presence of attenuated ASFV isolates in ASF-affected areas reduces the spreading of the virulent isolates and ASFV introduction into the domestic pig value chain. This identifies a need for future research into the evolution/stability of these low virulence ASFV isolates, molecular epidemiology, and immunology in sympatric populations of endemic areas. Knowledge concerning the role of gene deletion mutants in ASFV transmission among both wild and domestic compartments is also lacking and further investigation is, therefore, required.

## Data availability statement

The raw data supporting the conclusions of this article will be made available by the authors, without undue reservation.

## Ethics statement

The animal study was reviewed and approved by the Ethics Committee of the Complutense University of Madrid and the Community of Madrid (reference PROEX 159/19). The study was conducted in accordance with the local legislation and institutional requirements. No potentially identifiable images or data are presented in this study.

## Author contributions

Conceptualization, validation, and supervision: JS-V and JB. Methodology, writing—review and editing, and research: AK, LB, EC-F, SB-A, JB, and JS-V. Formal analysis, data cleansing, software, and visualization: AK, LB, and JB. Resources: JS-V. Writing—original draft preparation: AK and JB. All authors have read and agreed to the published version of the manuscript and contributed to the article and approved the submitted version.
